# Deletion of p38α MAPK in microglia blunts trauma-induced inflammatory responses in mice

**DOI:** 10.1186/s12974-019-1493-5

**Published:** 2019-05-10

**Authors:** Josh M. Morganti, Danielle S. Goulding, Linda J. Van Eldik

**Affiliations:** 1 Sanders-Brown Center on Aging, University of Kentucky, 101 Sanders-Brown Bldg., 800 S. Limestone Street, Lexington, KY 40536 USA; 2 Department of Neuroscience, University of Kentucky, Lexington, KY USA; 3 Spinal Cord and Brain Injury Research Center, University of Kentucky, Lexington, KY 40536 USA

**Keywords:** TBI, p38α, Kinase, Microglia, Monocytes, Cytokines, Chemokines, Neuroinflammation, Knockout

## Abstract

Traumatic brain injury (TBI) is a significant cause of morbidity and mortality in the USA and other developed countries worldwide. Following the initial mechanical insult, the brain’s primary innate immune effector, microglia, initiate inflammatory signaling cascades and pathophysiological responses that can lead to chronic neuroinflammation and neurodegenerative sequelae. The p38α MAPK signaling pathway in microglia is a key contributor to inflammatory responses to diverse disease-relevant stressors and injury conditions. Therefore, we tested here whether microglia p38α contributes to acute and persistent inflammatory responses induced by a focal TBI. We generated conditional cell-specific knockout of p38α in microglia using a CX3CR1 Cre-lox system, subjected the p38α knockout and wild-type mice to a controlled cortical impact TBI, and measured inflammatory responses at acute (1-day) and subacute (7-day) post-injury time points. We found that deletion of p38α in microglia only was sufficient to attenuate multiple pro-inflammatory responses following TBI, notably reducing pro-inflammatory cytokine/chemokine production and recruitment of inflammatory monocytes into the brain and preventing the persistent microglial morphological activation. These data provide strong evidence supporting a role for microglial p38α in propagation of a chronic and potentially neurotoxic pro-inflammatory environment in the brain following TBI.

## Introduction

Following TBI, the innate immune system is activated to induce the recruitment of microglia to the site of injury [1, 2], followed by the subsequent recruitment and infiltration of multiple waves of systemic immune mediators [3–6]. Presumably, these conserved tissue responses [7] are geared to protect and repair the brain from further injury. However, TBI can also lead to the persistent activation of microglia for many years following the initial TBI [8–10], and these secondary inflammatory responses can contribute to neurodegenerative sequelae [9–11].

A well-established signaling pathway that responds to a variety of inflammatory stressors is the p38 mitogen-activated protein kinase (MAPK) pathway, especially the p38α isoform [12]. Although p38α is expressed in virtually all cell types and tissues, it can have a variety of roles in development, homeostasis, and disease mechanisms. We have shown that p38α in microglia is a key component regulating the production of cytokines in response to a variety of disease-related stimuli in cell and animal models. For example, our previous findings using the LysM-Cre model of p38α deletion showed a robust suppression of trauma-induced inflammatory responses and protection against synaptic protein loss and vestibulomotor deficits [13]. However, a recent study [14] using the LysM-Cre system showed that LysM-driven recombination is not specific to only myeloid cell populations, but that there is a significant expression in neurons. Given the ubiquitous nature of p38α and recent reports of effects of neuronal p38α in animal models of degenerative disease [15–18], we sought to test more specifically the role of p38α in microglia following focal TBI.

Therefore, we generated a more microglia-specific p38α conditional knockout mouse, driven by the CX3CR1-Cre that can be used to selectively delete p38α from the brain’s resident immune cell, microglia. We tested if microglia p38α is a significant contributor to TBI-induced neuroinflammatory responses, including the production of pro-inflammatory cytokines and recruitment of peripheral monocytes into the brain. We report here that loss of p38α in microglia significantly reduces TBI-induced neuroinflammatory responses at both acute (1-day) and subacute (7-day) time points after injury.

## Methods

### Animals

All procedures involving animals for this study were approved by the Institutional Animal Care and Use Committee of the University of Kentucky. Mice were group housed in environmentally controlled conditions and provided food and water ad libitum.

To generate a cell-specific reporter line for validation of tamoxifen-induced recombination, we crossed homozygous CX3CR1^CreERT2^ mice [19] (Jax # 020940) with homozygous Gt (ROSA)26Sor^tm9(CAG-tdTomato)Hze^ (Ai9). Resultant heterozygous mice, *Ai9*^*ΔCX3CR1CreERT2*^, were used to measure tdTomato (RFP) expression in the brain myeloid fraction and in circulating peripheral blood mononuclear cells (PBMCs), following tamoxifen induction, as described below.

To generate the microglia-specific conditional knockout mice, we used our p38α^fl/fl^ mice [13] crossed with CX3CR1^CreERT2^ mice [19] (Jax # 020940) to generate hemizygous CX3CR1^+/CreERT2^p38α^+/fl^ mice. Hemizygous carriers were mated to p38α^fl/fl^ mice to generate CX3CR1^+/CreERT2^p38α^fl/fl^ mice (KO, knockout). To generate experimental cohorts, we mated the KO mice back to p38α^fl/fl^ mice to generate a roughly 50:50 distribution of Cre^+^p38α^fl/fl^ (KO) and Cre^neg^ p38α^fl/fl^ (WT, wildtype) littermates. All mice were genotyped by Transnetyx. For all experiments, both male and female mice were used in a 50:50 ratio.

*Ai9*^*ΔCX3CR1CreERT2*^, WT, and KO mice, 2 months old, were fed tamoxifen in chow (Harlan, #TD.130860, 400 ppm) for 28 days [19] to initiate Cre-mediated recombination in the KO mice as well as control for any confounds due to tamoxifen exposure in the WT mice. Following this period, there was a 28-day washout period wherein mice were fed standard diet, yielding experimental mice that were approximately 4 months old. The washout period was used because this Cre + mouse line has been shown to equally affect both microglia and bone marrow-derived mononuclear cells following tamoxifen administration [19]. However, following at least a 2-week washout of tamoxifen, peripheral mononuclear cells are replaced with non-recombined cells [19], whereas the recombined microglia persist.

### Surgery

Approximately 4-month-old WT and p38α KO mice underwent surgery for moderate focal TBI using the controlled cortical impact (CCI) method to the right parietal lobe, as we have previously described [5, 20, 21]. Briefly, mice were anesthetized, heads shaved, and maintained with 2.5% isoflurane with a non-rebreathing nose cone and passive exhaust system connected to a stereotaxic frame (Stoelting). Once animals were secured with non-traumatic ear bars, eye ointment was applied, and their shaved heads were swabbed with betadine. A midline incision was made through the scalp. Mice received a craniectomy approximately 3.5 mm in diameter using an electric microdrill with the center point determined by a digitally calibrated manipulator arm (Leica) to the coordinates: anteroposterior, − 2.0 mm; mediolateral, +2.0 mm, with respect to bregma. Explicit attention was paid to prevent damage to the dura during craniectomy; any animal in which the dura was disrupted, as assessed by excessive bleeding, was omitted from the study and replaced by another littermate. Following craniectomy, contusion was achieved using a 3.0-mm convex tip attached to an electromagnetic impactor (Leica) mounted to the digitally calibrated manipulator arm. The manipulator arm was rotated 20° on the vertical axis, to allow impact flush with the natural curvature of the head/tissue. The parameters for impact were for a contusion depth of 0.9 mm (from dura), velocity was constant at 4.0 m/s, and the impact was sustained for 300 ms. Importantly, these injury parameters penetrated all layers of the cortex stopping short of disrupting the dorsal hippocampal structure (AP − 2.0 mm; ML +2.0 mm; DV − 0.9 mm). Following CCI injury, the scalp was stapled, and each mouse was placed on a heating pad until they fully recovered as exhibited by resumption of movement and grooming. Sham animals were treated to the above surgical parameters except that the CCI injury was omitted. Animals were randomly allocated to their respective endpoint analyses at either 1-day or 7-day post-surgery.

### qRT-PCR

Following washout (described above) and euthanasia, enriched myeloid fraction was processed for RNA isolation using the RNeasy Plus mini kit (Qiagen #74136) following the manufacturer’s protocol. Eluted RNA was quantified using a NanoDrop 2000 (Thermo). Four hundred nanograms of eluted RNA was converted to cDNA using High Capacity cDNA Reverse Transcription Kit (Applied Biosystems #4368813). Quantitative Real-Time PCR for gene expression was performed on a ViiA7 Real-Time PCR System (Applied Biosystems) using TaqMan gene expression assays for *MAPK14* (Mm00442497_m1) and *HPRT* (Mm00446968_m1). Relative gene expression ratios were calculated using the 2^−ΔΔCT^ method. All data were Log_2_ transformed.

### MSD multiplex ELISA

At the designated interval, mice were anesthetized with 2.5% isoflurane and transcardially perfused with ice-cold phosphate-buffered saline (PBS) for 5 min. Following perfusion, the brains were rapidly removed, and the ipsilateral dorsal hippocampus was dissected and snap frozen in a 2-mL screw-top tube in liquid nitrogen. All dissected hippocampi were stored at − 80 °C for subsequent biochemical evaluation. Hippocampi were processed for protein extraction using a high shear homogenizer (Omni TH115) using lysis buffer at a 1:10 weight to volume ratio. Tissue lysis buffer consisted of PBS containing 1 mM PMSF and 1 mM EDTA. Hippocampal homogenate was centrifuged at 12,000×*g* for 20 min at 4 °C in a Heraeus Megafuge 16R. Supernatants were collected for measurement of cytokines and chemokines using MesoScale Discovery (MSD) custom multiplex high-sensitivity (V-Plex) ELISA kits, as we have previously described [13].

### Flow cytometry

For recombination validation using naïve Ai9 reporter mice, brains and blood from three sets of *Ai9*^*ΔCX3CR1CreERT2*^ mice were harvested following the 28d tamoxifen washout period and used for myeloid cell and PBMC isolation, respectively. Brain myeloid cells were enriched using a discontinuous Percoll gradient (30:70), as others and we have previously described [5, 22]. This method was also used in a naïve set of WT and KO mice to enrich myeloid cells from the brain to examine gene expression of p38α. Following centrifugation, the resultant myeloid enriched fraction at the 30:70 interface was aspirated and subsequently snap frozen prior to use for RNA harvesting and gene expression analyses.

PBMCs were isolated using diluted (1:3) fresh blood collected into plasma EDTA tubes, layered over FicollPaque+ (GE, #17-1440-02). PBMCs were enriched using centrifugation at 1500×*g* for 20mins at 4 °C. Cells were harvested and resuspended in FACS buffer (PBS + 0.5% FCS) for staining. Brain myeloid cells and PBMC’s were incubated with Zombie NIR (BioLegend, #423106) followed by Fc block (Miltenyi #130-092-575) and then anti-CD11b (BD #565976).

For examining trauma-induced infiltration of monocytes at 1 day following injury, mice were anesthetized with 2.5% isoflurane and transcardially perfused with ice-cold PBS for 5 min. Brains were rapidly removed and bisected, additionally removing the brainstem and cerebellum. The subsequent ipsilateral hemisphere was processed for myeloid cell enrichment using a discontinuous Percoll gradient, as described above. Following myeloid enrichment, cells were prepared for staining using Fc block (Miltenyi #130-092-575). Cell-surface labeling was conducted using conjugated antibodies against CD11b (BD #565976) and Ly6C (BD #560594). Inflammatory monocytes were defined by this sequential gating strategy to enumerate the CD11^+^Ly6C^+^ cell population. Cell viability was assessed using ZombieNIR (BioLegend #423105). Spectral compensation was achieved using polystyrene microparticles (Miltenyi #130-107-755) in combination with the above-listed antibodies following the manufacturer’s suggested protocol. Cell analysis was conducted on a BD LSR II flow cytometer and analyzed using FlowJo software (Treestar, v10.0).

### Immunohistochemistry and analysis

Using a sliding microtome with a freezing stage, we collected serial coronal sections (30 μm) of the ipsilateral hemi-brain through the entire hemisphere and stored the sections in cryoprotectant at − 20 °C. Staining procedures were conducted on free-floating sections using every 12th section through the entire hemisphere. Primary and secondary antibodies were diluted in 3% normal goat serum (LAMPIRE Biological Laboratories, catalog #7332500) with 0.2% Triton X-100. Endogenous peroxidase activity was quenched with 3% H_2_O_2_ in methanol, before the tissue blocking in 10% normal goat serum with 0.2% Triton X-100. Sections were incubated overnight at 4 °C with rabbit anti-Iba1 (1:10,000, Wako #019-19741), followed by incubation with a HRP-conjugated goat anti-rabbit (Vector) secondary antibody. Subsequently, sections were developed in 0.5 mg/ml 3,3-diaminobenzidine tetrahydrochloride solution (Sigma, catalog #D5637). The tissue sections were dehydrated through gradients of ethyl alcohol and finally xylene. The sections were coverslipped with Permount Mounting Medium (Fisher Scientific) and imaged on a Zeiss Axio Scan Z1 digital slide scanner at × 20 magnification. Image analysis of the ipsilateral peri-contusion cortex and dorsal hippocampus, done by an investigator blinded to the study, used Halo analysis suite (Indica Labs, v2.3.2089.34) with the positive pixel algorithm; AreaQuantification v1.0. Iba1 positive staining was set to a baseline threshold on WT sham Iba1^+^ microglia pixel intensity, such that any pixel at that intensity or greater (i.e., darker) was quantified as a pixel-positive area. The number of positive pixels was normalized per area outline for each section to account for outlined region-to-region area variability. All sections were batch analyzed using the same parameters.

### Statistics

Statistical analyses were done using GraphPad Prism (v8). Pre-planned contrasts to examine the effect of TBI in WT versus p38α KO were conducted using two-way ANOVA with Sidak multiple comparison correction to examine pairwise response of TBI cohorts. Statistical significance was defined as *p* < 0.05. All values are expressed as mean ± SEM.

## Results

### Microglial p38α knockout mitigates acute neuroinflammatory responses to TBI

**Fig. 1:**
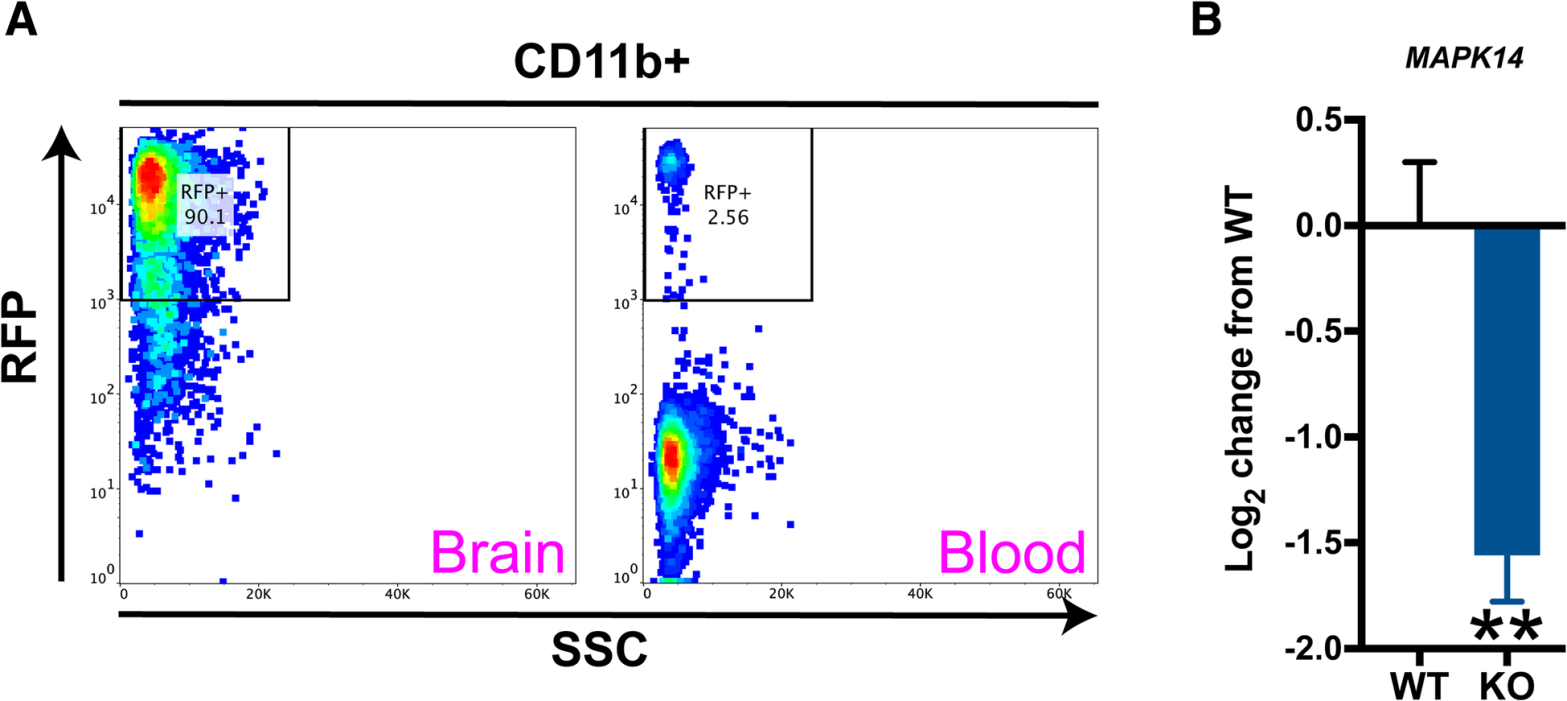
Validation of conditional microglial knockout of p38α. **a** A cell-specific reporter (*Ai9*^*ΔCX3CR1CreERT2*^) was used to validate the compartment restriction of tamoxifen-induced recombination. *Ai9*^*ΔCX3CR1CreERT2*^ mice were placed on tamoxifen chow for 28 days, followed by a washout of 28 days on regular chow. In naïve mice, brain myeloid and blood PBMCs were examined for tdTomato (RFP) expression in tandem with CD11b (BUV395) expression. After the 28 day washout, 90.1% of CD11b^+^ cells were labeled with RFP, whereas only 2.56% of circulating PBMCs were RFP^+^. **b** Myeloid-enriched fractions from naïve wild type (WT) and knockout (KO) mice were examined for p38α (*MAPK14*) gene expression following the tamoxifen washout procedures. Compared to WT mice, the p38α KO mice showed a significant reduction in p38α. Flow cytometry plots are representative of 3 separate experiments. For gene expression, *n* = 4 per group. Data were analyzed using Student’s *t* test, *p < 0.05. RFP red fluorescent protein, SSC side scatter

We validated the tissue restriction of the CX3CR1^CreERT2^ [19] line by crossing them to the Ai9 reporter line, inducing recombination with a 28-day induction with tamoxifen chow, and then using a 28-day washout period on standard chow to allow peripheral mononuclear cells to be replaced with non-recombined cells. As shown in Fig. 1a, this results in the overwhelming majority of tdTomato (RFP) expression being observed in the brain (90.1% of RFP^+^ CD11b^+^ cells in the brain versus 2.56% of RFP^+^ PBMCs in the blood). Further, using this induction followed by washout paradigm, we also found a significant decrease in p38α (*MAPK14*) gene expression in the enriched myeloid fraction from p38α KO mice compared to WT mice (Fig. 1b). These data document the microglial selectivity of the CX3CR1-Cre system and demonstrate a substantial decrease in p38α expression in the KO mice.

**Fig. 2:**
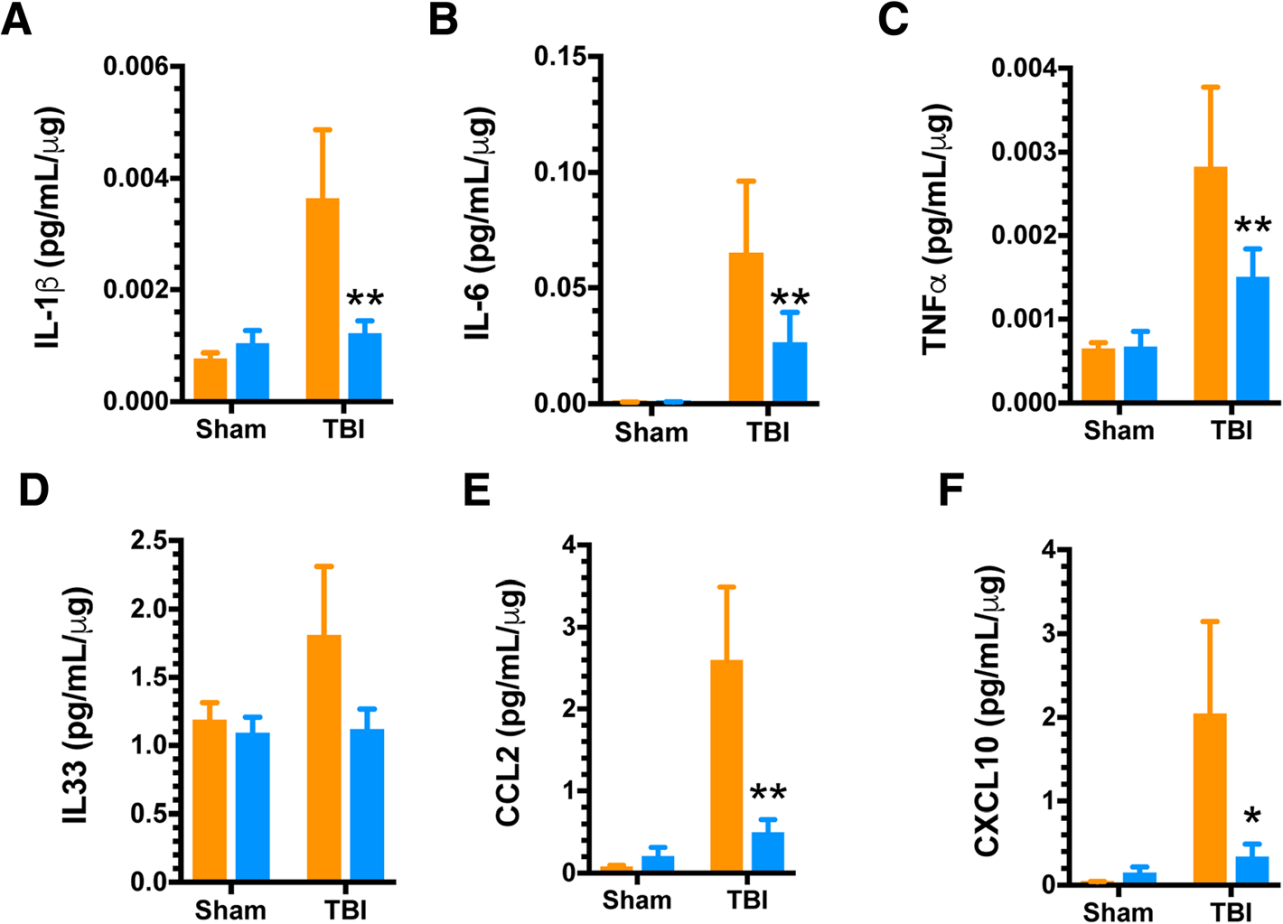
TBI-induced acute pro-inflammatory cytokine and chemokine up-regulation is mitigated by microglial p38α deficiency. Ipsilateral dorsal hippocampi were analyzed via high sensitivity multiplex MSD ELISA assay for pro-inflammatory cytokines and chemokines from sham and injured animals (*n* = 8–10/group) at a 1 day post-injury interval. **a**–**c** The TBI-induced increases in pro-inflammatory cytokines IL-1β, IL-6, and TNFα were significantly blunted, with ~ 50% lower levels in the p38α KO mice compared to WT mice. **d** The TBI-induced increase in IL-33 was also reduced in KO compared to WT mice, but this change was not significant. **e**,**f** The pro-inflammatory chemokines CCL2 and CXCL10 showed a marked reduction in the KO mice, compared to their injured WT counterparts. Data were analyzed using two-way ANOVA with Sidak’s multiple comparison corrections on the pre-planned contrasts examining the effect of TBI in the WT versus KO conditions. *p < 0.05, **p < 0.01, comparing KO (blue bars) to WT (orange bars)

We have previously shown that p38α is an integral mediator of microglial inflammatory responses in vitro and in vivo [23]. Further, p38α knockout in myeloid cells using the LysM-Cre model is neuroprotective following TBI [13]. However, because of recent data indicating significant expression in neurons with this LysM-Cre model system [14], it was important to test the effect of the more microglia-specific, CX3CR1-Cre mediated p38α deletion on trauma-induced pro-inflammatory responses. To do this, we first examined the acute response of multiple neuroinflammatory analytes in WT and p38α KO mice at 1-day post-injury. As shown in Fig. 2, CCI induces a robust increase in multiple pro-inflammatory cytokines and chemokines in the ipsilateral hippocampus, and these responses are significantly reduced in the p38α KO mice. Specifically, TBI-induced upregulation of the cytokines IL-1β, IL-6, and TNFα was significantly blunted in the KO mice (Fig. 2a–c), with a similar trend in IL-33 levels that did not reach significance (Fig. 2d). Similarly, there was a significant reduction in levels of the monocyte recruitment chemokines CCL2 (Fig. 2e) and CXCL10 (Fig. 2f) in the KO mice after TBI.

**Fig. 3:**
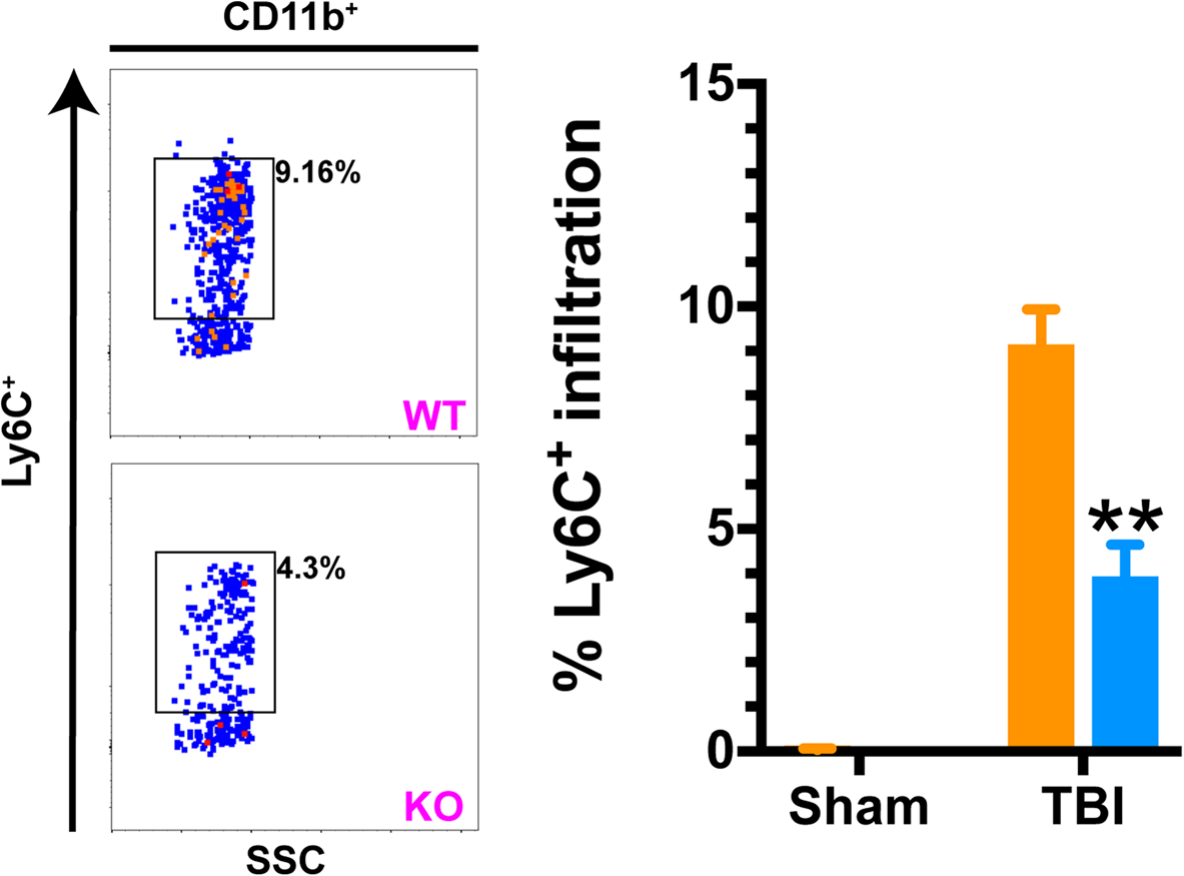
TBI-mediated recruitment of inflammatory monocytes is significantly reduced in mice lacking microglial p38α. Using flow cytometric analyses, we quantified the proportional change in cellular infiltrate into the ipsilateral hemisphere of mice 1 day following injury. Inflammatory monocytes were defined by a sequential gating strategy to enumerate CD11^+^Ly6C^+^ cell population in all four groups (WT and p38α KO, sham, and TBI). There were little-to-none inflammatory monocytes in the sham brains of either WT or KO mice. However, there was a robust increase observed in this cell population 1 day following TBI in WT mice, and this pathophysiological response was significantly blunted by ~ 50% in the KO mice. Data were analyzed using two-way ANOVA with Sidak’s multiple comparison corrections on the pre-planned contrasts examining the effect of TBI in the WT versus KO conditions. **p < 0.01, comparing KO (blue bars) to WT (orange bars). SSC side scatter

Given the significant decrease in multiple pro-inflammatory cytokines as well as chemotactic mediators of monocyte recruitment, we next examined whether p38α KO mice were spared from TBI-induced monocyte recruitment to the injured brain. We have recently shown that there is a temporally coordinated recruitment of inflammatory monocytes to the brain following TBI that peaks approximately 1 day post-injury [5, 20]. Therefore, we used this time point to quantify this response using flow cytometry with a combination of antigenic markers designed to delineate activated microglia from infiltrated monocytes. As shown in Fig. 3, we found that the p38α KO mice have approximately 50% less infiltration of peripherally derived monocytes into the injured brain, compared to WT mice.

### Removal of microglial p38α blunts subacute inflammatory and histopathological responses to TBI

**Fig. 4:**
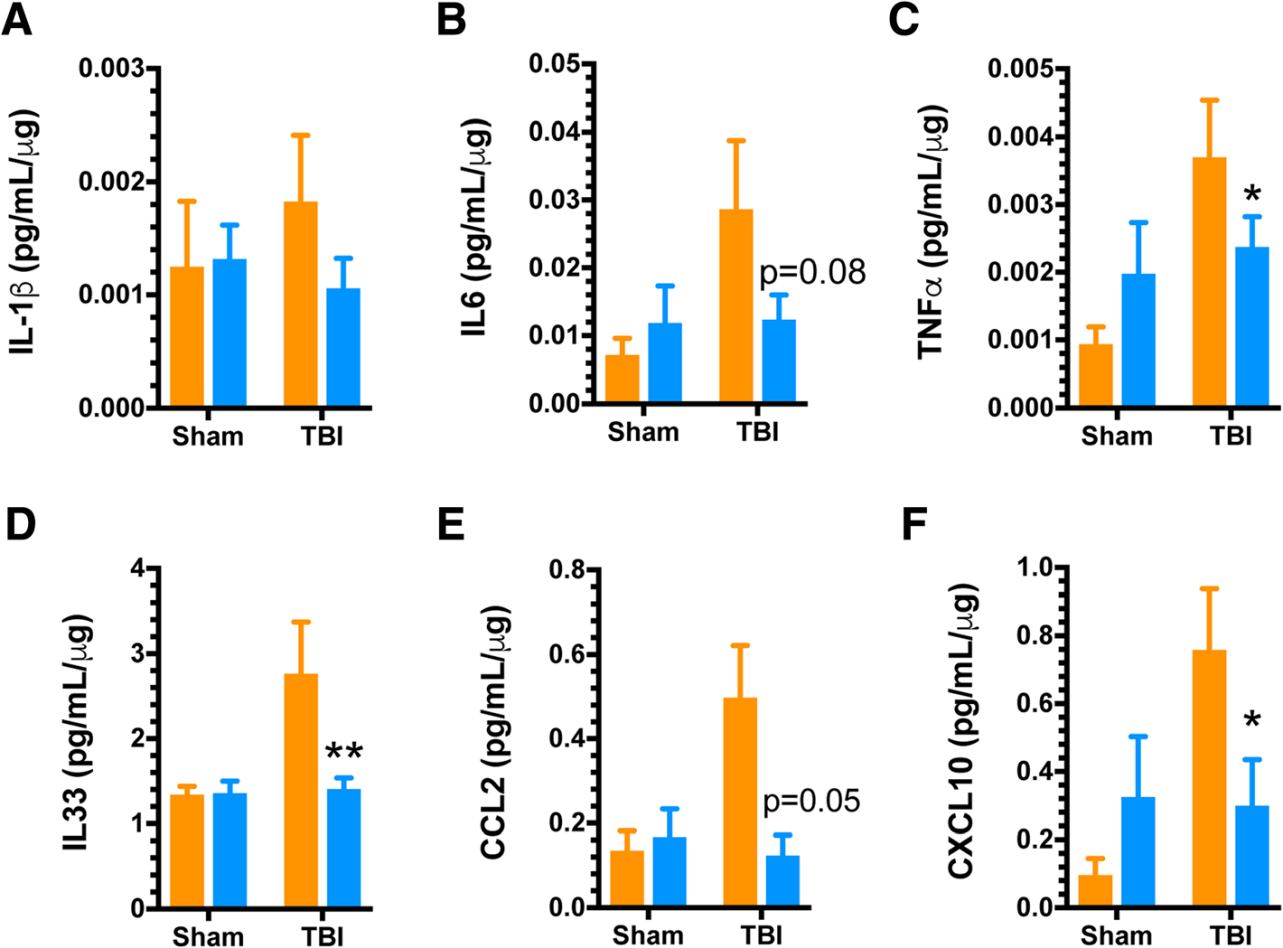
Persistent pro-inflammatory response is blunted by microglial p38α deletion. Ipsilateral dorsal hippocampi were analyzed via high sensitivity multiplex MSD ELISA assay for pro-inflammatory cytokines and chemokines from sham and injured animals (*n* = 8–10/group) at a 7 days post-injury interval. **a**, **b**Trauma-induced expression of IL-1β had subsided to approximately basal levels in all groups. There was a persistent trend for increased IL6 production in WT mice that was reduced in KO mice, but the values did not reach significance. **c**,**d** The chronic elevation of TNFα was significantly reduced in the KO compared to WT mice. The levels of IL-33 remained in an elevated state in WT mice but were significantly reduced in KO mice. **e**, **f** The chronic elevation of the chemokines CCL2 and CXCL10 seen in the WT mice was reduced in the KO mice. Data were analyzed using two-way ANOVA with Sidak’s multiple comparison corrections on the pre-planned contrasts examining the effect of TBI in the WT versus KO conditions. *p < 0.05, **p < 0.01, comparing KO (blue bars) to WT (orange bars)

**Fig. 5:**
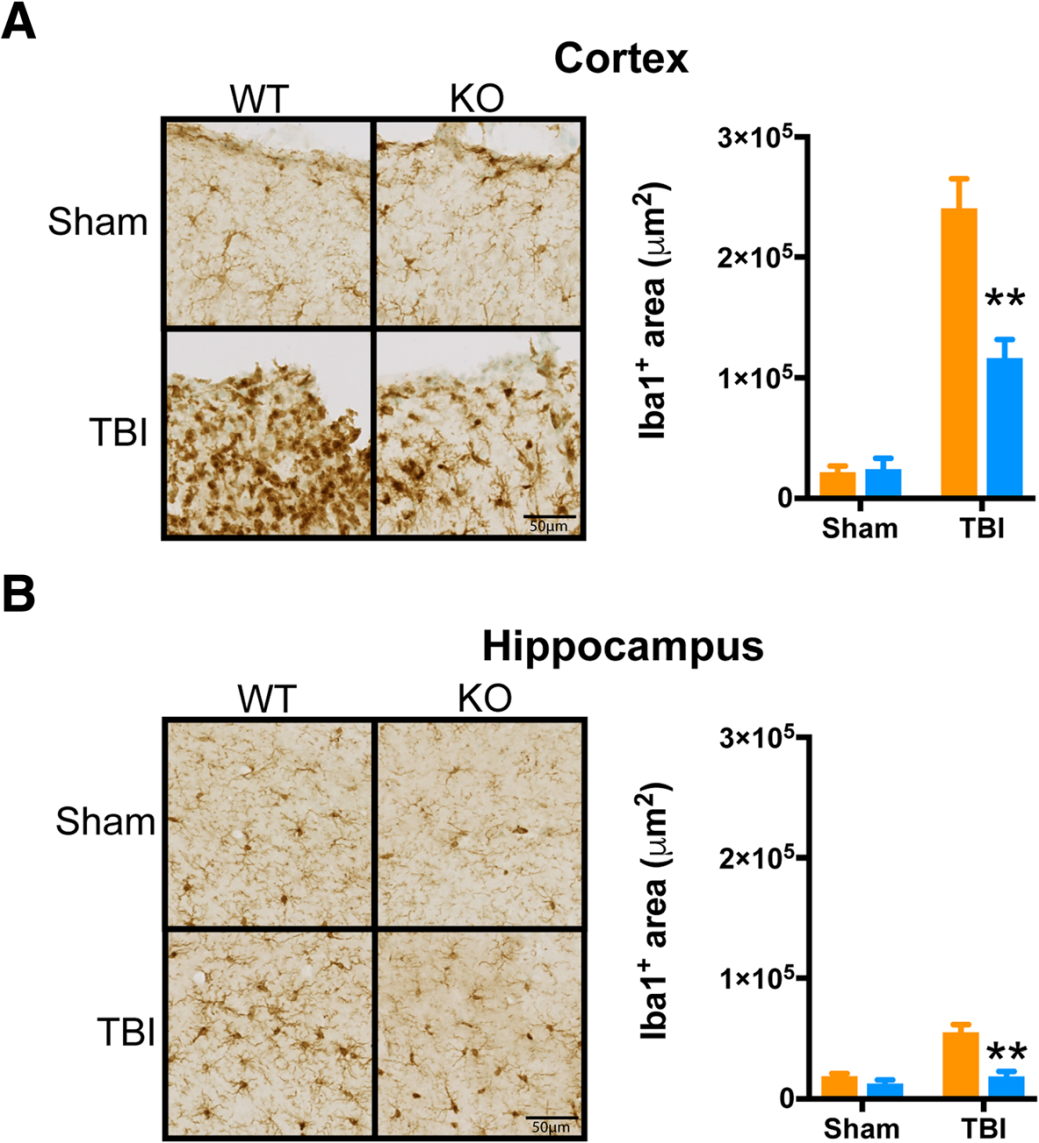
TBI-induced persistent alterations in microglial morphology are mitigated by knockout of p38α in microglia. Tissue sections spanning the ipsilateral peri-contusion cortex and dorsal hippocampal formation were quantified for the positive staining area of Iba1 at the 7 days post-injury interval. Using a pixel-positive algorithm, we observed a lessened reactivity with Iba1^+^ area in both the peri-contusion (**a**) and dorsal hippocampus CA1 molecular layer (**b**) for injured KO mice, relative to injured WT. Morphologically, Iba1 staining appeared to have increased amoeboid phenotype in the injured cortex of WT mice, relative to injured KO. Similarly, there was a visual trend for hippocampal microglia to have larger soma in injured WT mice, compared to KO. In both the cortex (**a**) and hippocampus (**b**), p38α KO mice subjected to TBI had a significantly reduced Iba1+ area fraction compared to their WT controls. Data were analyzed using two-way ANOVA with Sidak’s multiple comparison corrections on the pre-planned contrasts examining the effect of TBI in the WT versus KO conditions. **p < 0.01, comparing KO (blue bars) to WT (orange bars)

We next examined the consequences of the absence of p38α-driven microglial responses to trauma in the context of subacute inflammatory responses (7 days post-injury). This phase following injury represents a period where peripheral immune infiltration has stopped, and the bulk of inflammatory responses are associated with resident effectors, as we have previously described [24]. Therefore, we tested here the neuroinflammatory responses of WT and p38α KO mice at 7 days following injury (Fig. 4). At this post-injury interval, IL-1β had returned to approximately basal levels (Fig. 4a), while there was a non-significant trend for IL-6 reduction (Fig. 4b) in the KO mice. The KO mice had significantly blunted pro-inflammatory cytokine response for TNFα (Fig. 4c) and IL-33 (Fig. 4d), as well as a significant reduction in the pro-inflammatory chemokines CCL2 (Fig. 4e) and CXCL10 (Fig. 4f). We also examined the histopathological response of microglia in the dorsal hippocampus at this subacute interval. Using Iba1 as a marker of microglia/macrophage morphology, we observed a protracted increase in Iba1+ area in the peri-contusion cortex and the dorsal hippocampus of WT mice at 7 days post-injury. However, this persistent TBI-induced microglial response was significantly blunted by approximately 50% in the peri-contusion cortex and entirely absent in the p38α KO mouse at this time point (Fig. 5a, b).

## Discussion

In the current study, we have demonstrated a microglia-specific role for p38α in the production of trauma-induced neuroinflammatory sequelae at acute and subacute intervals. We showed that p38α plays an integral role in the production of multiple pro-inflammatory signaling mediators and potentiates the recruitment of inflammatory monocytes to the injured parenchyma. Moreover, our current results demonstrate that deletion of p38α from microglia provides significant amelioration of both acute and protracted trauma-induced inflammatory responses, including accelerating the return of microglia morphological phenotypes towards basal phenotypes. Collectively, these findings further demonstrate that microglial p38α plays an integral role in propagating trauma-induced neuroinflammatory sequelae.

Microglia have an intrinsic ability to mobilize and rapidly respond to injury [1, 2]. However, the unabated and/or chronic activation of these cells is an integral link in the pathophysiological response propagating dysfunction sequelae, in the clinical setting as well as in preclinical animal models of TBI trauma [25–28]. In terms of the involvement of p38-mediated inflammatory responses in CNS trauma, multiple reports have documented a significant role for p38 following both TBI and spinal cord injury [29–32]. Mice that are deficient in MK2, which is a direct and selective substrate of p38α, showed significant improvements in multiple measures of recovery following spinal cord injury, compared to WT [30]. Collectively, there is growing data demonstrating a role for p38α and its regulated effectors in promoting dysfunctional responses following CNS trauma.

Our results demonstrate that deletion of p38α in microglia provides significant amelioration of whole-tissue pro-inflammatory cytokines and chemokines and significantly limits the ingress of inflammatory monocytes that we have previously attributed to causing significant detrimental effects upon neuronal and cognitive function [5]. Therefore, our new findings specifically implicate microglial p38α as an integral mediator creating a permissive environment for the recruitment of neurotoxic monocytes.

Further, the effects of targeting p38α in microglia provided protracted benefits, as there was a collective mitigation of trauma-induced pro-inflammatory bias in KO animals relative to their WT counterparts at a subacute 7-day post-injury interval. These effects were seen by the return of microglia morphological response to sham-like levels in the KO mice, compared to WT mice, which still exhibited a perturbed morphological phenotype.

Our current findings warrant extended exploration of the role of p38α in microglia responses to TBI. It still remains unknown if the alterations in pro-inflammatory bias conferred by loss of p38α in microglia offer neuroprotective capacities and whether these ultimately offer functional restoration at chronic intervals.

## Conclusion

Restraint of microglia and their inflammatory responses following neurotrauma represents an ever-growing area for targeted therapeutic development. Our current efforts further support the potential benefits of discretely targeting microglia through inhibition of p38α, or potentially its downstream intermediates, as a potential strategy to blunt neuroinflammatory sequelae.

## Abbreviations

CCI: Controlled cortical impact
KO: Knockout
MAPK: Mitogen-activated protein kinase
MSD: MesoScale Discovery
PBMCs: Peripheral blood mononuclear cells
PBS: Phosphate-buffered saline
RFP: Red fluorescent protein
TBI: Traumatic brain injury
W: Wildtype
